# Controlling Nutritional Status score as a predictor of ventricular arrhythmias in patients with advanced heart failure

**DOI:** 10.1093/eschf/xvag037

**Published:** 2026-02-26

**Authors:** Yuma Ono, Hidekazu Kondo, Taisuke Harada, Kunio Yufu, Hiroki Sato, Kazuki Mitarai, Keisuke Yonezu, Katsunori Tawara, Akira Fukui, Hidefumi Akioka, Yasushi Teshima, Naohiko Takahashi

**Affiliations:** Department of Cardiology and Clinical Examination, Faculty of Medicine, Oita University, 1-1 Idaigaoka, Hasama, Yufu City, Oita 879-5593, Japan; Department of Cardiology and Clinical Examination, Faculty of Medicine, Oita University, 1-1 Idaigaoka, Hasama, Yufu City, Oita 879-5593, Japan; Department of Cardiology, NHO Oita Medical Center, Oita City, Oita, Japan; Department of Cardiology, Oita Red Cross Hospital, Oita City, Oita, Japan; Department of Cardiology and Clinical Examination, Faculty of Medicine, Oita University, 1-1 Idaigaoka, Hasama, Yufu City, Oita 879-5593, Japan; Department of Cardiology and Clinical Examination, Faculty of Medicine, Oita University, 1-1 Idaigaoka, Hasama, Yufu City, Oita 879-5593, Japan; Department of Cardiology and Clinical Examination, Faculty of Medicine, Oita University, 1-1 Idaigaoka, Hasama, Yufu City, Oita 879-5593, Japan; Department of Cardiology and Clinical Examination, Faculty of Medicine, Oita University, 1-1 Idaigaoka, Hasama, Yufu City, Oita 879-5593, Japan; Department of Cardiology and Clinical Examination, Faculty of Medicine, Oita University, 1-1 Idaigaoka, Hasama, Yufu City, Oita 879-5593, Japan; Department of Cardiology and Clinical Examination, Faculty of Medicine, Oita University, 1-1 Idaigaoka, Hasama, Yufu City, Oita 879-5593, Japan; Department of Advanced Medical Sciences, Faculty of Medicine, Oita University, Yufu City, Oita, Japan; Department of Cardiology and Clinical Examination, Faculty of Medicine, Oita University, 1-1 Idaigaoka, Hasama, Yufu City, Oita 879-5593, Japan

**Keywords:** Cardiac resynchronization therapy, CONUT score, Heart failure, Malnutrition, Mortality, Ventricular arrhythmia

## Abstract

**Background and Aims:**

Malnutrition is common among heart failure (HF) patients and may affect prognosis. Its effects on arrhythmia outcomes in HF patients remain unclear. We evaluated whether malnutrition, as assessed using the Controlling Nutritional Status (CONUT) score and Geriatric Nutritional Risk Index (GNRI), predicts ventricular arrhythmias and all-cause mortality in advanced HF patients receiving cardiac resynchronization therapy (CRT).

**Methods:**

This retrospective single-centre cohort study enrolled 167 patients (mean age 70.9 ± 9.5 years, 67.1% male) who underwent CRT between March 2004 and February 2023. Nutritional status was assessed using the CONUT score and GNRI before CRT. Malnutrition was defined as a CONUT score ≥5 and a GNRI score <92. The primary endpoint was a composite of ventricular arrhythmias and all-cause mortality. The median follow-up period was 1536 days (IQR: 844–1825 days).

**Results:**

Malnutrition was identified in 26 patients (15.6%) based on CONUT scores and in 37 patients (22.2%) based on GNRI scores, showing moderate agreement (*κ* = 0.44). Kaplan–Meier survival analysis demonstrated significantly higher event rates in patients with CONUT-defined malnutrition for the primary outcome (log-rank *P* = .0003). Conversely, GNRI-defined malnutrition exhibited only a weak trend (log-rank *P* = .06). When examined separately, both nutritional indices predicted all-cause mortality (CONUT: *P* = .0001; GNRI: *P* = .01), whereas only CONUT-defined malnutrition significantly predicted ventricular arrhythmias (CONUT: *P* = .01; GNRI: *P* = .38). The multivariate Cox regression analysis confirmed CONUT-defined malnutrition as an independent predictor of the primary outcome (adjusted HR: 2.33, 95% CI: 1.30–4.20, *P* < .01). Adding the CONUT score to the base model significantly improved discrimination (concordance index: 0.695 to 0.713, *P* = .008). Time-dependent receiver operating characteristic analysis showed an AUC of 0.80 (95% CI: 0.67–0.94) at 1825 days for CONUT-defined malnutrition.

**Conclusions:**

CONUT-defined malnutrition was a strong independent predictor of ventricular arrhythmias and all-cause mortality in CRT recipients. Nutritional assessment may enhance risk stratification in patients with advanced HF undergoing CRT.

## Graphical Abstract

**Graphical Abstract:**
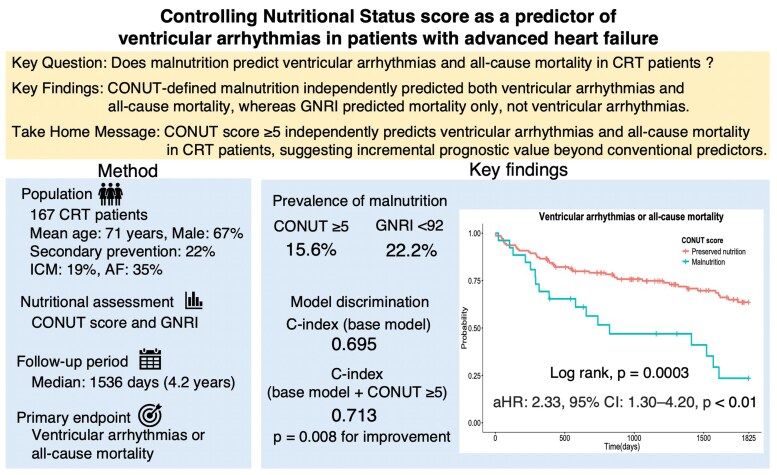

## Introduction

Malnutrition is highly prevalent among older adults owing to age-related physiological decline and comorbidities.^1^ Concurrently, heart failure (HF) is becoming increasingly common among older populations owing to an ageing society. Cardiovascular deaths are more prevalent among patients with HF and reduced ejection fraction (HFrEF), particularly from worsening HF and ventricular arrhythmias, whereas non-cardiovascular deaths predominate in patients with preserved ejection fraction (EF).^2^ Guideline-directed medical therapy remains the cornerstone of HFrEF management;^3^ however, for patients with wide QRS complexes and poor response to pharmacotherapy, cardiac resynchronization therapy (CRT) offers a viable option for improving functional status and reducing morbidity and mortality.^4–6^

Malnutrition is associated with adverse outcomes in patients with HF. Nutritional decline can result from multiple mechanisms, including intestinal oedema, reduced food intake, chronic inflammation, and catabolic states. Moreover, malnutrition-associated inflammation may lead to myocardial remodelling and contribute to arrhythmogenesis. Although several studies have established the prognostic impact of malnutrition in HF,^7,8^ the evidence remains limited to populations with advanced HF undergoing CRT, particularly regarding the prediction of ventricular arrhythmic events.

Therefore, this study aimed to assess the prevalence of malnutrition using the Controlling Nutritional Status (CONUT) score and Geriatric Nutritional Risk Index (GNRI) and to evaluate the association between malnutrition and clinical outcomes, including ventricular arrhythmias and all-cause mortality, among patients with advanced HF treated with CRT.

## Methods

### Study design and patient population

**Figure 1:**
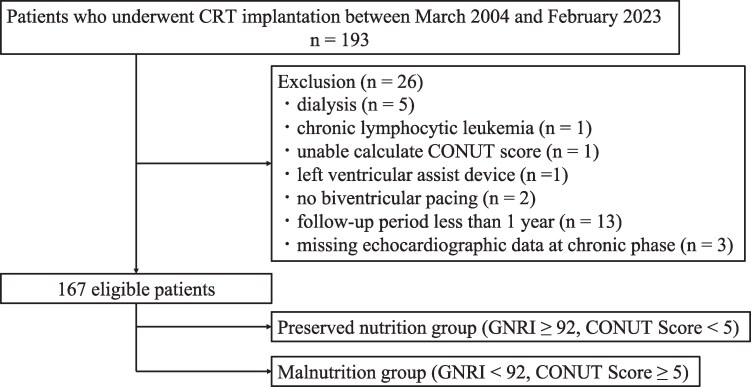
Study protocol. CRT, cardiac resynchronization therapy; CONUT, Controlling Nutritional Status; GNRI, Geriatric Nutritional Risk Index

This was a retrospective single-centre cohort study. The study protocol is illustrated in *Figure 1*. Between March 2004 and February 2023, we enrolled 193 patients who underwent CRT. The exclusion criteria were as follows: patients on dialysis (*n* = 5), patients with chronic lymphocytic leukaemia (*n* = 1), patients whose CONUT score could not be calculated (*n* = 1), patients with a left ventricular assist device (*n* = 1), patients without left ventricular pacing (*n* = 2), patients with a follow-up period of less than 1 year (*n* = 13), and patients with missing echocardiographic data during the chronic phase (*n* = 3). Hence, 167 patients were eligible for the study. The patients were divided into two groups according to their nutritional status, which was assessed prior to CRT device implantation. We followed up with the patients for at least 1 year and up to 5 years after CRT device implantation. The indications for CRT adhered to the Japanese Circulation Society’s guidelines. All procedures were performed in accordance with the principles outlined in the 1964 Declaration of Helsinki, and the study was approved by the Oita University Ethics Committee (Approval number 3068). Informed consent was obtained from all the participants through an opt-out system.

### Nutritional assessment

The Global Leadership Initiative on Malnutrition criteria are globally standardized for diagnosing malnutrition.^9^ As this was a retrospective cohort study, nutritional assessment was performed using the CONUT score and the GNRI. The CONUT score is a nutritional evaluation tool reported by Ignacio *et al.*^10^ It was calculated based on the serum albumin (Alb) level, total cholesterol level, and total lymphocyte count. Nutritional status was classified into four categories according to the CONUT score: normal (0–1), mild (2–4), moderate (5–8), and severe (9–12). The GNRI is another nutritional evaluation tool developed by Bouillanne *et al.*^11^ It was calculated using the following formula: 1.489 × serum Alb (g/L) + 41.7 × (body weight in kg/ideal body weight). The ideal body weight was calculated using the following formula: 22 × height squared in metres. Nutritional status was classified into four categories according to the GNRI: major risk (GNRI <82), moderate risk (GNRI: 82– < 92), low risk (GNRI: 92–98), and no risk (GNRI > 98). These nutritional assessment tools are commonly used in studies of patients with HF. The cut-off value for the CONUT score was defined as 5, and that for the GNRI was 92, as these cut-off values are associated with HF prognosis.^7,12^

### Definition of CRT response

CRT responders were defined as patients with at least a 15% reduction in the left ventricular end-systolic volume (LVESV) at least 6 months after undergoing CRT device implantation.^13^ Patients were classified as non-responders if they met any of the following criteria: readmission for HF, worsening of the New York Heart Association (NYHA) class at the final follow-up, continuous moderate-to-severe deterioration of the clinical composite score^14^ at the final medical examination, permanent discontinuation of biventricular pacing due to worsening HF, or death within 6 months after CRT.^13^

### Echocardiographic assessment

Echocardiographic measurements were performed using a 1.5–4.0 MHz transducer at an appropriate depth on apical and parasternal views. M-mode, two-dimensional, and Doppler examinations were performed. LVESV, left ventricular end-diastolic volume, and left ventricular EF were evaluated using the modified biplane Simpson’s rule.

### Study outcomes

Information on the patients’ clinical outcomes was retrospectively obtained from their medical records. The primary endpoint was a composite of ventricular arrhythmia events and all-cause mortality. The secondary endpoints were ventricular arrhythmia and all-cause mortality. Ventricular arrhythmias were defined as ventricular tachycardia (VT) or ventricular fibrillation (VF) requiring anti-tachycardia pacing or cardioversion. Among these events, only the first event was considered in the statistical analysis.

### Statistical analysis

Normally distributed continuous variables are presented as means ± standard deviation, and non-normally distributed variables are presented as medians with interquartile ranges. Categorical variables are presented as numbers and percentages. The Bazett correction formula (QTcB) was used to correct QT. In patients with hypoalbuminemia, adjusted calcium values were calculated using Payne’s formula: adjusted calcium value = actual calcium value + (4 − Alb value). The total number of comorbidities had an interquartile range from 3 to 5. Comorbidities included hypertension, dyslipidaemia, diabetes mellitus, chronic obstructive pulmonary disease, atrial fibrillation, chronic kidney disease (estimated glomerular filtration rate [eGFR] < 60 mL/min/1.73 m2), anaemia (haemoglobin [Hb] < 13 g/dL for males and <12 g/dL for females, based on the World Health Organization criteria), history of cerebrovascular disease, history of percutaneous coronary intervention or coronary artery bypass grafting, history of peripheral artery disease, and history of cancer. Student’s *t*-test and Wilcoxon test were used for continuous variables, and the chi-squared test or Fisher’s exact test were used for categorical variables. Survival was estimated using the Kaplan–Meier method and compared using the log-rank test. Univariate and multivariate Cox proportional hazards regression analyses were performed to identify the factors predicting the primary outcome. Age, sex, eGFR, left bundle branch block (LBBB), median brain natriuretic peptide (BNP), N-terminal pro-brain natriuretic peptide (NT-proBNP) levels (BNP: 352.6 pg/mL, NT-proBNP: 1759 pg/mL), and history of ventricular arrhythmias were used in the multivariate analysis. To evaluate the incremental predictive value of malnutrition, we added CONUT score ≥5 to the base model (including MADIT-ICD benefit score, LBBB, eGFR, median BNP and NT-proBNP levels, and history of ventricular arrhythmias) and evaluated model discrimination using Harrell’s concordance index (CI) and log-likelihood ratio (LLR).^15^ Time-dependent ROC curve analysis was performed to evaluate the predictive performance of the CONUT score for the primary outcome at 1825 days. The time-dependent area under the curve (AUC) was calculated using the inverse probability of the censoring weighting method for handling right-censored data. Time-dependent ROC curve analysis was adjusted for covariates, including LBBB, median BNP and NT-proBNP levels, and history of ventricular arrhythmias.

To explore the relationship between the CONUT score and hazard ratio (HR) for the primary outcome, a restricted cubic spline analysis with three knots was performed. The analysis was conducted using a CONUT score of 1 as the reference value, which corresponds to the cut-off value for normal nutrition. The restricted cubic spline model was adjusted for covariates, including LBBB, median BNP, and NT-proBNP levels, and history of ventricular arrhythmias.

The results are presented as hazard ratios (HRs) with 95% confidence intervals (CIs). Statistical significance was set at a two-tailed *P*-value of less than .05.

All statistical analyses were performed using EZR version 1.68 (Saitama Medical Center, Jichi Medical University, Saitama, Japan), which is a graphical user interface for R version 4.4.1 (The R Foundation for Statistical Computing, Vienna, Austria).^16^ The figures were generated using R.

## Results

### Patient characteristics

**Figure 2:**
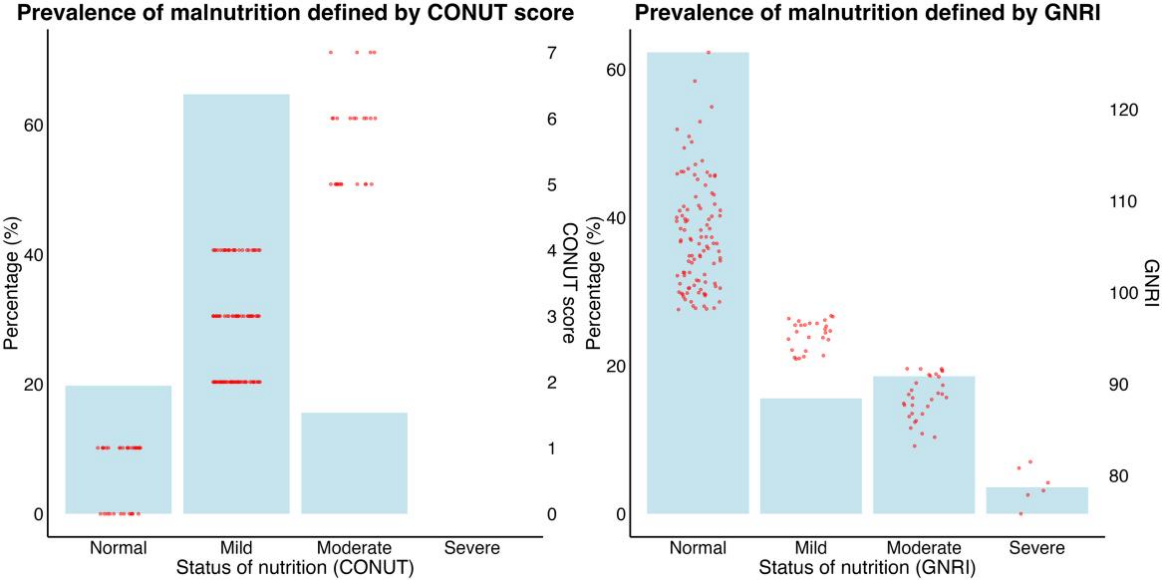
Prevalence of nutritional disorders defined by the CONUT score and GNRI in the study participants. CONUT, Controlling Nutritional Status; GNRI, Geriatric Nutritional Risk Index

Among the 167 patients, the mean age was 70.9 ± 9.5 years. Of these patients, 112 (67.1%) were male, 32 (19.2%) had ischaemic cardiomyopathy, and 37 (22.2%) were undergoing secondary prevention. Additionally, 59 (35.3%) patients had atrial fibrillation, and 83 (49.7%) were classified as NYHA functional class III or IV. The mean EF was 31.4% ± 8.5%, and 114 (68.3%) patients exhibited LBBB. The mean QRS duration was 165 ± 26 ms, and 93 (55.7%) patients were CRT responders. The baseline patient characteristics are shown in *Table 1*. The prevalence of malnutrition, as defined by the CONUT score and GNRI in eligible patients is shown in *Figure 2*. A total of 26 (15.6%) and 37 patients (22.2%) had malnutrition according to their CONUT score (≥5) and GNRI (< 92), respectively. These nutritional assessments showed moderate agreement (*κ* = 0.44; 95% CI: 0.25–0.62).

**Table 1:** Baseline characteristics of the malnutrition and preserved nutrition groups defined by the CONUT score and GNRI

		CONUT score		*P*	GNRI		*P*
	Total cohort (*n* = 167)	Preserved nutrition (*n* = 141)	Malnutrition (*n* = 26)		Preserved nutrition (*n* = 130)	Malnutrition (*n* = 37)	
Age (years)	70.9 ± 9.5	70.5 ± 9.4	72.9 ± 9.9	.23	70.3 ± 9.1	73.1 ± 10.6	.11
Age ≥75 years (%)	65 (38.9%)	51 (36.2%)	14 (53.8%)	.12	45 (34.6%)	20 (54.1%)	.**04**
Sex, male	112 (67.1%)	93 (66.0%)	19 (73.1%)	.65	86 (66.2%)	26 (70.3%)	.70
Body height (cm)	159.2 ± 10.3	159.4 ± 9.6	158.3 ± 13.4	.62	159.9 ± 9.6	156.7 (12.1)	.09
Body weight (kg)	58.0 ± 11.9	58.3 ± 10.8	56.5 ± 16.8	.46	60.9 ± 11.0	48.1 ± 9.6	**<**.**01**
Body mass index (kg/m²)	22.8 ± 3.8	22.9 ± 3.4	22.3 ± 5.3	.43	23.8 ± 3.5	19.5 ± 2.4	**<**.**01**
Systolic blood pressure (mmHg)	108.7 ± 16.8	109.8 ± 16.4	102.7 ± 18.0	**<**.**05**	109.9 ± 16.0	104.4 ± 19.0	.08
Heart rate (b.p.m.)	69.4 ± 15.1	69.0 ± 14.8	71.5 ± 16.8	.42	68.6 ± 14.6	72.0 ± 16.5	.22
Primary prevention	130 (77.8%)	112 (79.4%)	18 (69.2%)	.30	102 (78.5%)	28 (75.7%)	.82
MADIT-ICD benefit score							
Lowest	50 (29.9%)	39 (27.7%)	11 (42.3%)	.26	32 (24.6%)	18 (48.6%)	**0**.**02**
Intermediate	91 (54.5%)	78 (55.3%)	13 (50.0%)		76 (58.5%)	15 (40.5%)	
Highest	26 (15.6%)	24 (17.0%)	2 (7.7%)		22 (16.9%)	4 (10.8%)	
VT/VF score	6.5 ± 2.3	6.5 ± 2.3	6.7 ± 2.4	.69	6.5 ± 2.3	6.3 ± 2.4	.61
Score ≥ 7	81 (48.5%)	70 (49.6%)	11 (42.3%)	.53	65 (50.0%)	16 (43.2%)	.58
Non-arrhythmic mortality score	3.4 ± 2.0	3.3 ± 2.0	3.9 ± 1.9	.15	3.1 ± 2.0	4.5 ± 1.7	**<**.**01**
Score ≥3	105 (62.9%)	85 (60.3%)	20 (76.9%)	.13	75 (57.7%)	30 (81.1%)	.**01**
ICD benefit score	46.6 ± 22.5	47.4 ± 22.4	42.2 ± 22.5	.28	48.5 ± 21.9	39.9 ± 23.4	.**04**
CRT-D	136 (81.4%)	116 (82.3%)	20 (76.9%)	.58	112 (86.2%)	24 (64.9%)	.**01**
NYHA							
II	84 (50.3%)	80 (56.7%)	4 (15.4%)	**<**.**01**	73 (56.2%)	11 (29.7%)	.**01**
III	67 (40.1%)	52 (36.9%)	15 (57.7%)		48 (36.9%)	19 (51.4%)	
IV	16 (9.6%)	9 (6.4%)	7 (26.9%)		9 (6.9%)	7 (18.9%)	
Comorbidity	3 [3, 5]	3 [2, 4]	4 [3, 5]	.06	3 [2, 5]	3 [3, 5]	.91
Hypertension	58 (34.7%)	48 (34.0%)	10 (38.5%)	.66	47 (36.2%)	11 (29.7%)	.56
Diabetes	52 (31.1%)	40 (28.4%)	12 (46.2%)	.11	41 (31.5%)	11 (29.7%)	1.00
Dyslipidemia	77 (46.1%)	66 (46.8%)	11 (42.3%)	.83	62 (47.7%)	15 (40.5%)	.46
Chronic obstructive pulmonary disease	8 (4.8%)	4 (2.8%)	4 (15.4%)	.**02**	2 (1.5%)	6 (16.2%)	**<**.**01**
Atrial fibrillation	59 (35.3%)	50 (35.5%)	9 (34.6%)	1.00	48 (36.9%)	11 (29.7%)	.44
Chronic kidney disease (eGFR < 60 mL/min/1.73 m^2^)	131 (78.4%)	107 (75.9%)	24 (92.3%)	.07	98 (75.4%)	33 (89.2%)	.11
Anemia (male < 13 g/dL, female < 12 g/dL)	79 (47.3%)	59 (41.8%)	20 (76.9%)	**<**.**01**	55 (42.3%)	24 (64.9%)	.**02**
History of cerebrovascular disease	17 (10.2%)	16 (11.3%)	1 (3.8%)	.48	16 (12.3%)	1 (2.7%)	.12
History of VT/VF	37 (22.2%)	29 (20.6%)	8 (30.8%)	.30	28 (21.5%)	9 (24.3%)	.82
History of NSVT	78 (46.7%)	65 (46.1%)	13 (50.0%)	.83	62 (47.7%)	16 (43.2%)	.71
History of AMI	25 (15.0%)	19 (13.5%)	6 (23.1%)	.23	17 (13.1%)	8 (21.6%)	.20
History of PCI or CABG	59 (35.3%)	50 (35.5%)	9 (34.6%)	1.00	48 (36.9%)	11 (29.7%)	.44
History of hospitalization for heart failure	116 (69.5%)	96 (68.1%)	20 (76.9%)	.49	86 (66.2%)	30 (81.1%)	.11
History of PAD	12 (7.2%)	9 (6.4%)	3 (11.5%)	.40	8 (6.2%)	4 (10.8%)	.30
History of cancer	25 (15.0%)	20 (14.2%)	5 (19.2%)	.55	19 (14.6%)	6 (16.2%)	.80
Etiology of HF							
DCM	70 (41.9%)	59 (41.8%)	11 (42.3%)	1.00	57 (43.8%)	13 (35.1%)	.45
ICM	32 (19.2%)	26 (18.4%)	6 (23.1%)	.59	25 (19.2%)	7 (18.9%)	1.00
HCM	5 (3.0%)	5 (3.5%)	0	1.00	4 (3.1%)	1 (2.7%)	1.00
Sarcoidosis	28 (16.8%)	24 (17.0%)	4 (15.4%)	1.00	23 (17.7%)	5 (13.5%)	.63
Valvular disease	10 (6.0%)	8 (5.7%)	2 (7.7%)	.66	6 (4.6%)	4 (10.8%)	.23
Amyloidosis	8 (4.8%)	6 (4.3%)	2 (7.7%)	.61	6 (4.6%)	2 (5.4%)	1.00
Others without amyloidosis	14 (8.4%)	13 (9.2%)	1 (3.8%)	.70	9 (6.9%)	5 (13.5%)	.20
Laboratory data							
Hb (g/dL)	12.7 ± 1.7	12.8 ± 1.7	12.0 ± 1.7	.**03**	12.9 ± 1.7	11.9 ± 1.5	.**002**
C-reactive protein (mg/dL)	0.12 [0.06, 0.34]	0.10 [0.05, 0.22]	0.44 [0.26, 1.38]	**<**.**01**	0.1 [0.1, 0.3]	0.3 [0.1, 0.6]	.**003**
Alb (g/dL)	3.8 ± 0.5	4.0 ± 0.4	3.2 ± 0.3	**<**.**01**	4.0 ± 0.4	3.4 ± 0.4	**<**.**01**
T-chol (mg/dL)	167.5 ± 41.9	169.5 ± 40.7	157.1 ± 47.2	.17	169.0 ± 42.2	162.5 ± 40.9	.41
Lymphocyte count (/μL)	1407.7 ± 583.4	1477.0 ± 588.1	1031.9 ± 386.4	**<**.**01**	1468.3 ± 603.1	1194.8 ± 453.8	.**01**
Na (mEq/L)	139.2 ± 3.6	139.4 ± 3.1	138.1 ± 5.8	.10	139.7 ± 3.0	137.4 ± 5.0	**<**.**01**
K (mEq/L)	4.3 ± 0.5	4.3 ± 0.5	4.1 ± 0.6	.10	4.4 ± 0.5	4.2 ± 0.6	.**04**
Ca (mg/dL)	9.4 ± 0.4	9.4 ± 0.4	9.5 ± 0.3	.08	9.3 ± 0.4	9.6 ± 0.5	**<**.**01**
eGFR (mL/min/1.73m²)	49.1 ± 20.5	50.5 ± 20.7	41.2 ± 17.5	.**03**	49.9 ± 21.2	46.2 ± 18.0	.34
BNP (pg/mL)	352.6 [165.8, 676.3]	335.0 [140.8, 591.8]	485.0 [317.1, 835.0]	.07	333.2 [131.0, 572.6]	440.7 [291.5, 866.0]	.**04**
NT-BNP (pg/mL)	1759.0 [820.0, 3539.8]	1705.0 [788.7, 3524.0]	2719.0 [1949.5, 3824.0]	.10	1666.0 [783.3, 3407.0]	3500.0 [2260.0, 5077.0]	**<**.**01**
CONUT score	3 [2, 4]	2 [2, 3]	6 [5, 6]	**<**.**01**	2 [2, 3]	4 [3, 6]	**<**.**01**
GNRI score	100.3 ± 9.6	102.2 ± 8.3	89.8 ± 9.7	**<**.**01**	104.0 ± 6.9	86.9 ± 4.3	**<**.**01**
Echocardiographic data							
Ejection fraction (%)	31.4 ± 8.5	31.4 ± 8.4	31.2 ± 9.1	.92	31.4 ± 8.5	31.4 ± 8.8	.99
LVEDV (mL)	161.7 ± 62.2	160.7 ± 63.0	166.8 ± 58.2	.65	162.6 ± 62.2	158.6 ± 62.7	.73
LVESV (mL)	112.9 ± 51.4	112.2 ± 52.3	116.8 ± 46.8	.68	11f3.4 ± 51.2	111.1 ± 52.4	.81
QRS duration (ms)	163.8 ± 28.9	163.7 ± 28.8	164.2 ± 29.7	.94	162.8 ± 29.9	167.2 ± 24.9	.42
QTc (ms)	500 ± 40	500 ± 40	490 ± 40	.45	500 ± 40	500 ± 30	.83
Long QTc (male > 470 ms, female > 480 ms)	121 (72.9%)	103 (73.6%)	18 (69.2%)	.64	92 (71.3%)	29 (78.4%)	.53
LBBB	114 (68.3%)	98 (69.5%)	16 (61.5%)	.49	89 (68.5%)	25 (67.6%)	1.00
Responder	93 (55.7%)	81 (57.4%)	12 (46.2%)	.29	74 (56.9%)	19 (51.4%)	.58
Medications at discharge							
Heart failure medication	3 [2, 3]	3 [2, 3]	3 [2, 3]	.44	3 [2, 3]	2 [2, 3]	.14
ACEi or ARB or ARNI	136 (81.4%)	116 (82.3%)	20 (76.9%)	.58	108 (83.1%)	28 (75.7%)	.34
ACEi	65 (38.9%)	57 (40.4%)	8 (30.8%)	.39	50 (38.5%)	15 (40.5%)	.85
ARB	56 (33.5%)	44 (31.2%)	12 (46.2%)	.18	43 (33.1%)	13 (35.1%)	.85
ARNI	16 (9.6%)	16 (11.3%)	0 (0.0%)	.08	16 (12.3%)	0	.**02**
B-blocker	151 (90.4%)	127 (90.1%)	24 (92.3%)	1.00	117 (90.0%)	34 (91.9%)	1.00
MRA	110 (65.9%)	93 (66.0%)	17 (65.4%)	1.00	86 (66.2%)	24 (64.9%)	1.00
SGLT2i	28 (16.8%)	26 (18.4%)	2 (7.7%)	.26	25 (19.2%)	3 (8.1%)	.14
Diuretics							
Loop diuretics	107 (64.1%)	88 (62.4%)	19 (73.1%)	.38	82 (63.1%)	25 (67.6%)	.70
Tolvaptan	34 (20.4%)	24 (17.0%)	10 (38.5%)	.**02**	21 (16.2%)	13 (35.1%)	.**02**
Thiazide	2 (1.2%)	1 (0.7%)	1 (3.8%)	.29	1 (0.8%)	1 (2.7%)	.40
Inotropic agents							
Pimobendane	28 (16.8%)	20 (14.2%)	8 (30.8%)	**<**.**05**	19 (14.6%)	9 (24.3%)	.21
Digoxin	10 (6.0%)	7 (5.0%)	3 (11.5%)	.19	6 (4.6%)	4 (10.8%)	.23
Antiarrhythmics							
Amiodarone	84 (50.3%)	70 (49.6%)	14 (53.8%)	.83	64 (49.2%)	20 (54.1%)	.71
Antiarrhythmics other than amiodarone	11 (6.6%)	8 (5.7%)	3 (11.5%)	.38	8 (6.2%)	3 (8.1%)	.71
Antiplatelet medications	43 (25.7%)	34 (24.1%)	9 (34.6%)	.33	33 (25.4%)	10 (27.0%)	.83
Anticoagulant medications	89 (53.3%)	78 (55.3%)	11 (42.3%)	.29	65 (50.0%)	24 (64.9%)	.14
CCB	17 (10.2%)	15 (10.6%)	2 (7.7%)	1.00	13 (10.0%)	4 (10.8%)	1.00
HMG-CoA reductase inhibitors (Statins)	84 (50.3%)	71 (50.4%)	13 (50.0%)	1.00	68 (52.3%)	16 (43.2%)	.36
Ezetimibe	6 (3.6%)	5 (3.5%)	1 (3.8%)	1.00	5 (3.8%)	1 (2.7%)	1.00
Prednisolone	18 (10.8%)	14 (9.9%)	4 (15.4%)	.49	14 (10.8%)	4 (10.8%)	1.00

Bold type indicates statistical significance. ACEi, angiotensin-converting enzyme inhibitor; Alb, albumin; AMI, acute myocardial infarction; ARB, angiotensin II receptor blocker; ARNI, angiotensin receptor-neprilysin inhibitor; BNP, brain natriuretic peptide; CABG, coronary artery bypass grafting; CCB, calcium channel blocker; CONUT, controlling nutrition status; CRT-D, cardiac resynchronization therapy-defibrillator; DCM, dilated cardiomyopathy; eGFR, estimated glomerular filtration rate; GNRI, geriatric nutritional risk index; Hb, haemoglobin; HCM, hypertrophic cardiomyopathy; HF, heart failure; ICD, implantable cardioverter-defibrillator; ICM, ischaemic cardiomyopathy; LBBB, left bundle branch block; LVEDV, left ventricular end-diastolic volume; LVESV, left ventricular end-systolic volume; MRA, mineralocorticoid receptor antagonist; NSVT, non-sustained ventricular tachycardia; NYHA, New York Heart Association Functional Classification; NT-proBNP, N-terminal pro-brain natriuretic peptide; PAD, peripheral arterial disease; PCI, percutaneous coronary intervention; QTc, corrected QT interval; SGLT2i, sodium-glucose cotransporter 2 inhibitor; T-chol, total cholesterol; VF, ventricular fibrillation; VT, ventricular tachycardia.

The comparison of baseline characteristics between the patients with CONUT-defined/GNRI-defined malnutrition and those without is also shown in *Table 1*. Malnutrition, as defined by the CONUT score, was associated with lower blood pressure, NYHA class III/IV disease, chronic obstructive pulmonary disease, and anaemia. CONUT-defined malnutrition was associated with lower Hb and Alb levels, lymphocyte counts, eGFR, and higher C-reactive protein levels. Regarding medications, higher tolvaptan and pimobendane use was associated with CONUT-defined malnutrition. The associations of malnutrition, as defined by the GNRI, showed some similarities with those defined by the CONUT score, although some differences were observed. A higher prevalence of GNRI-defined malnutrition among patients aged more than 75 years and with lower body weight and body mass index was specifically observed. Patients classified as malnourished according to the GNRI had a higher MADIT non-arrhythmic mortality score, were more frequently categorized in the lowest MADIT-implantable cardioverter-defibrillator (ICD) benefit score group, and were less likely to receive a CRT-defibrillator (D). Regarding biomarkers, lower levels of sodium (Na) and potassium (K) and higher levels of C-reactive protein, calcium (Ca), BNP, and NT-proBNP were significantly associated with malnutrition, as defined by the GNRI. Only a lack of angiotensin receptor-neprilysin inhibitor therapy was significantly associated with medication use.

### Clinical outcomes

The median follow-up period was 1536 days (interquartile range: 844–1825 days). During follow-up, ventricular arrhythmias and all-cause mortality were observed in 61 patients. Of these, 35 had ventricular arrhythmias, 39 died from any cause, and 13 had both outcomes. All-cause mortality was due to cardiovascular causes in 26 patients (66.7%), infection in 4 (10.3%), cancer in 2 (5.1%), and other or unknown causes in 5 (12.8%). No inappropriate device activation was observed.

**Figure 3:**
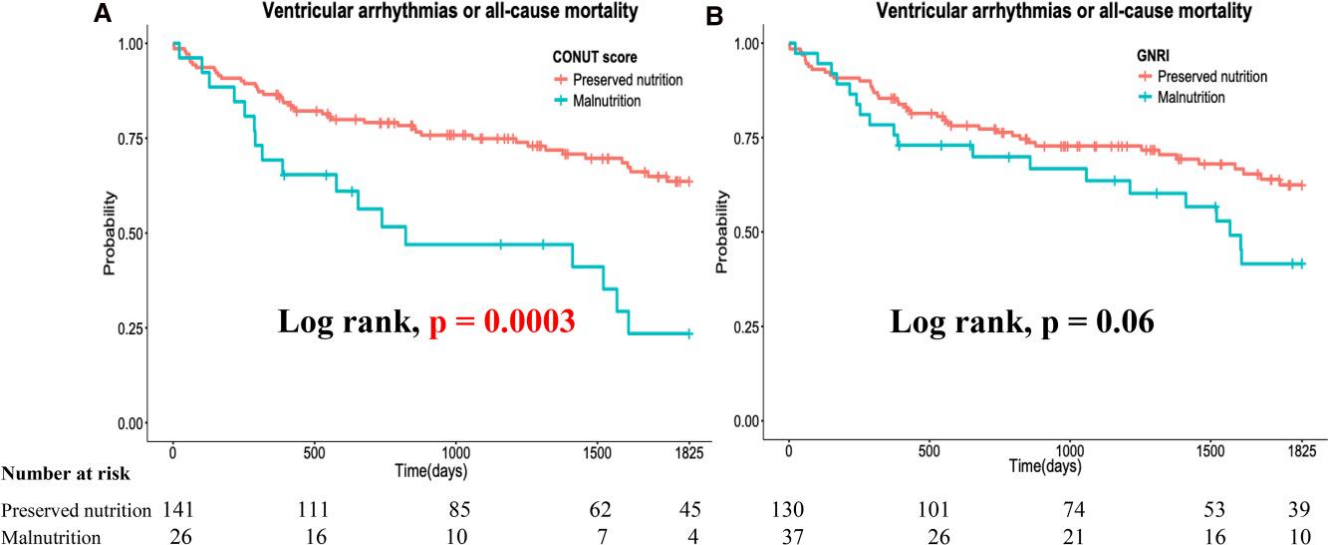
Kaplan–Meier survival analysis and log-rank test for the primary endpoint stratified by the CONUT score (*A*) and GNRI (*B*). CONUT, Controlling Nutritional Status; GNRI, Geriatric Nutritional Risk Index

**Figure 4:**
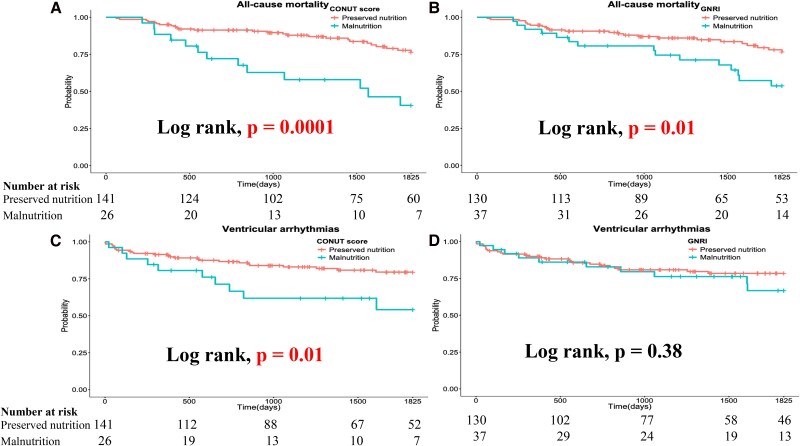
Kaplan–Meier survival analysis and log-rank test for the secondary endpoints stratified by the CONUT score and GNRI: (*A*) All-cause mortality stratified by the CONUT score, (*B*) All-cause mortality stratified by the GNRI, (*C*) Ventricular arrhythmias stratified by the CONUT score, and (*D*) Ventricular arrhythmias stratified by the GNRI. CONUT, Controlling Nutritional Status; GNRI, Geriatric Nutritional Risk Index

**Figure 5:**
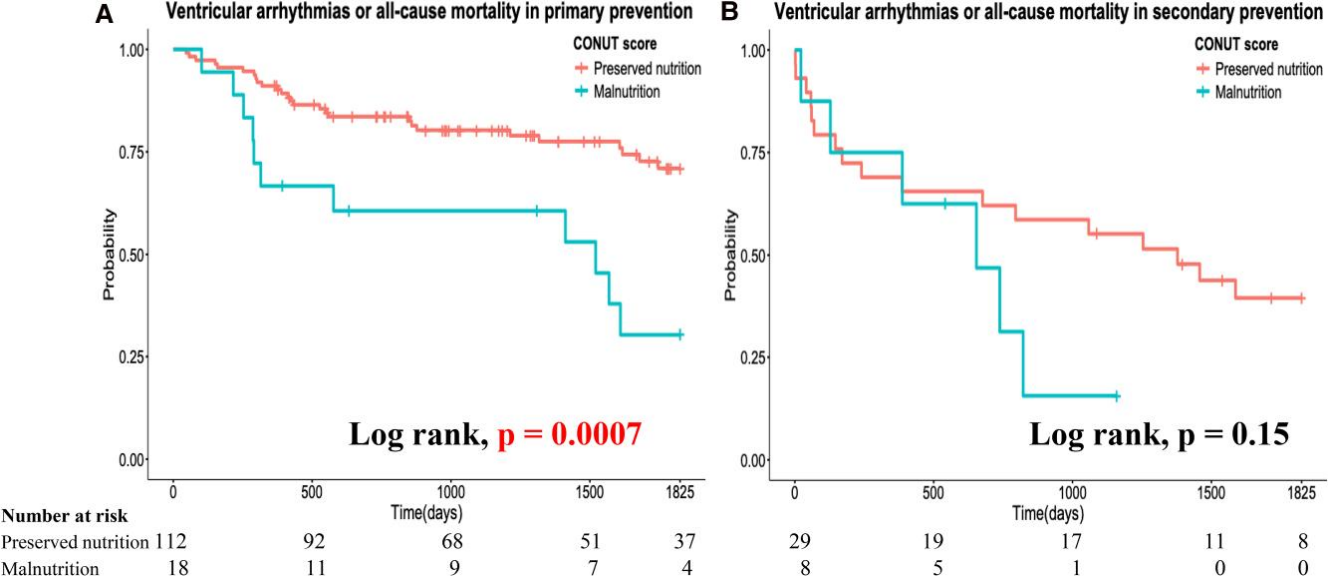
Kaplan–Meier survival analysis and log-rank test for the primary endpoint stratified by the CONUT score in primary prevention and secondary prevention: (*A*) primary endpoint in the primary prevention, (*B*) primary endpoint in the secondary prevention. CONUT, Controlling Nutritional Status

The primary outcome was significantly higher among patients with malnutrition as defined by the CONUT score than in those without malnutrition (65.3% vs 31.2%, respectively; *P* = .002). Malnourished patients according to the GNRI showed a trend towards a higher primary outcome, although this did not reach statistical significance (51.4% vs 32.3%, respectively; *P* = .052). Regarding secondary outcomes, all-cause mortality was significantly higher among patients with malnutrition according to both criteria (CONUT score: 50.0% vs 18.4%, *P* = .002; GNRI: 40.5% vs 18.5%, *P* = .008). However, ventricular arrhythmia was significantly more frequent among patients with malnutrition as defined by the CONUT score (38.5% vs 17.7%; *P* = .03), whereas no significant difference was observed among those defined by the GNRI (27.0% vs 19.2%; *P* = .36). Kaplan–Meier survival analysis and log-rank test showed that patients with malnutrition, as defined by the CONUT score, had a significantly higher event rate for the primary outcome (log-rank: *P* = .0003). In contrast, patients with malnutrition, as defined by the GNRI, exhibited an increased event rate, but this difference was not statistically significant (log-rank test: *P* = .06) (*Figure 3A* and *B*). Patients with malnutrition also had a significantly higher all-cause mortality event rate (CONUT score: log-rank, *P* = .0001; GNRI: log-rank, *P* = .01) (*Figure 4A* and *B*). However, ventricular arrhythmias were significantly more frequent among patients with malnutrition as defined by the CONUT score, whereas no significant difference was observed among those defined by the GNRI (CONUT score: log-rank, *P* = .01; GNRI: log-rank, *P* = .38) (*Figure 4C* and *D*). Furthermore, a significantly higher event rate was observed among patients with malnutrition, as defined by the CONUT score, only for primary prevention (*Figure 5A*, *P* = .0007), whereas no significant difference was observed for secondary prevention (*Figure 5B*, *P* = .15). Univariate and multivariate Cox proportional hazard analyses showed that malnutrition, as defined by the CONUT score, was significantly associated with a higher incidence of the primary outcome, whereas this was not the case for malnutrition, as defined by the GNRI (*Table 2*). The addition of malnutrition defined by CONUT scores ≥5 to the base model significantly improved model performance in predicting the primary outcome (*Table 3*). The base model alone achieved a concordance index (CI) of 0.695. When malnutrition was added to the base model, the CI increased to 0.713 with an LLR improvement of −3.54 (*P* = .008), indicating significant enhancement in model discrimination.

**Table 2:** Event rate and univariate and multivariate cox proportional hazards analyses of clinical outcomes

	Preserved malnutrition	Malnutrition	Univariate Cox regression analysis		Multivariate Cox regression analysis			
			HR (95% CI)	*P*	Model 1	*P*	Model 2	*P*
	Event rate/100 patient-years	Event rate/100 patient-years			Adjusted HR (95% CI)		Adjusted HR (95% CI)	
Using CONUT score								
Primary outcome; ventricular arrhythmias or all-cause mortality	8.42	22.18	2.74 (1.56–4.81)	<.01	2.82 (1.60–4.96)	<.01	2.33 (1.30–4.20)^a^	<.01
Secondary outcome; all-cause mortality	4.98	16.96	3.42 (1.75–6.67)	<.01	3.17 (1.62–6.20)	<.01	2.94 (1.48–5.84)^a^	<.01
Secondary outcome; ventricular arrhythmias	4.78	13.05	2.43 (1.16–5.06)	.02	2.56 (1.23–5.37)	.01	1.82 (0.84–3.95)^b^	.13
Using GNRI								
Primary outcome; ventricular arrhythmias or all-cause mortality	8.96	14.56	1.68 (0.98–2.89)	.06	1.77 (1.02–3.06)	.04	1.55 (0.90–2.69)^a^	.12
Secondary outcome; all-cause mortality	5.12	11.49	2.24 (1.18–4.28)	.01	2.13 (1.11–4.08)	.02	2.00 (1.03–3.88)^a^	.04
Secondary outcome; ventricular arrhythmias	5.33	7.66	1.39 (0.67–2.90)	.38	1.54 (0.73–3.22)	.25	1.35 (0.64–2.85)^b^	.43

CI, confidence interval; CONUT, Controlling Nutritional Status; GNRI, Geriatric Nutritional Risk Index; HR, hazard ratio.

Model 1: adjusted for age and sex.

^a^ Model 2: adjusted for left bundle branch block (LBBB), estimated glomerular filtration rate (eGFR), median brain natriuretic peptide (BNP), N-terminal pro-brain natriuretic peptide (NT-proBNP) levels, and history of ventricular arrhythmias.

^b^ Model 2: adjusted for age, sex, LBBB, median BNP, NT-proBNP level, and history of ventricular arrhythmias.

**Table 3:** Addition of malnutrition defined by CONUT score to base model improves model performance in predicting primary outcome

Model	Concordance index	LLR improvement from base	*P* value for LLR improvement from base
Base model	0.695		
Base model + Malnutrition (CONUT Score ≥ 5)	0.713	−3.54	.008

LLR, log-likelihood ratio; CONUT, Controlling Nutritional Status.

Variables adjusted in the base model: MADIT-ICD benefit score, left bundle branch block (LBBB), estimated glomerular filtration rate (eGFR), median brain natriuretic peptide (BNP) and N-terminal pro-brain natriuretic peptide (NT-proBNP), and history of ventricular arrhythmias.

Improvement in model performance was measured using Harrell’s concordance index (CI) and LLR: the more negative the LLR, the bigger the improvement in model performance, as described previously.^17^

Time-dependent ROC curve analysis after adjustment for covariates (LBBB, median BNP and NT-proBNP levels, and history of ventricular arrhythmias) showed that the AUC for malnutrition defined by the CONUT score (≥5) in predicting the primary outcome was 0.80 (95% CI: 0.67–0.94) at 1825 days (Supplementary Figure S1). To further explore the relationship between the CONUT score and HR of the primary outcome, we conducted a restricted cubic spline analysis with three knots, using a CONUT score of 1 as the reference. The multivariable-adjusted restricted cubic spline analysis with three knots demonstrated a significant association between the CONUT score and the hazard of the primary outcome (overall *P* = .03). The test for non-linearity was not significant (*P* = .78).

## Discussion

This study had several novel findings. First, among patients with advanced HF undergoing CRT, 15.6% were classified as malnourished based on a CONUT score of 5 or more, and 22.2% using a GNRI of less than 92. Second, CONUT-defined malnutrition was a significant predictor of poor clinical outcomes, including both ventricular arrhythmias and all-cause mortality. In contrast, GNRI-defined malnutrition was associated with mortality but not with fatal ventricular arrhythmias. Third, the CONUT score demonstrated high prognostic accuracy with a time-dependent AUC of 0.80 at 5 years and significantly improved risk stratification when added to conventional predictors (CI improved from 0.695 to 0.713, *P* = .008), offering incremental prognostic value in CRT candidates.

The prevalence of malnutrition among patients with HF varies widely across studies and is influenced by differences in the assessment tools, cut-off values, and patient characteristics. Hu *et al.* reported malnutrition rates ranging from 6% to 73% among patients with HF depending on the method used.^8^ Sze *et al.* demonstrated that GNRI and CONUT score had poor concordance for mild malnutrition but greater concordance for moderate-to-severe malnutrition in stable outpatients with chronic HF.^17^

In this study, moderate-to-severe malnutrition was observed in 15.6% (CONUT) and 22.2% (GNRI) of patients. Our restricted cubic spline analysis demonstrated a linear relationship between the CONUT score and the risk of the primary outcome. The test for non-linearity was not significant (*P* = .78). Additionally, our study validated the utility of a CONUT score cut-off of 5, which has been used in previous HF studies, for predicting outcomes in CRT patients. This cut-off value may serve as a practical threshold for risk stratification in this population, consistent with previous data from chronic HF cohorts.

Given that the patients were in a stable condition rather than the acute phase, our baseline characteristic analysis revealed that C-reactive protein levels were higher in patients with malnutrition defined by the CONUT score and GNRI compared to those with preserved nutrition. This suggests that elevated C-reactive protein levels reflect chronic inflammation rather than acute confounding factors (such as infection). Malnutrition is associated with poor prognosis in HF patients. Previous studies have demonstrated an association between malnutrition and chronic inflammation. Elevated levels of inflammatory cytokines, such as tumour necrosis factor-α (TNF-α), interleukin (IL)-1, and IL-6, have been observed in patients with chronic HF.^18^ These inflammatory cytokines target the central nervous system to induce anorexia and promote muscle catabolism, leading to sarcopenia.^19,20^ Thus, they contribute to malnutrition through the combined effects of decreased energy intake and increased energy expenditure. Given this association between malnutrition and inflammation, assessment tools that capture both aspects may be clinically valuable. The three components of the CONUT score (serum albumin, total cholesterol, and lymphocyte count) are influenced by both nutritional status and immune activity.^19,20^ In contrast, GNRI depends on weight and serum albumin and is a more nutrition-specific indicator. Importantly, our findings demonstrate that CONUT-defined malnutrition not only correlates with mortality but also with the occurrence of ventricular arrhythmias, a relationship not observed with the GNRI. This finding suggests that it is not malnutrition alone, but rather the malnutrition-inflammation complex, that is important for arrhythmia development.

The mechanisms linking malnutrition to ventricular arrhythmias are complex. Lazzerini *et al.* reported that inflammatory activation is increasingly recognized as a non-conventional risk factor for arrhythmias. In their review, they demonstrated that inflammatory cytokines (such as TNF-α, IL-1, and IL-6) can promote ventricular arrhythmias through multiple direct and indirect mechanisms. These include myocardial fibrosis, conduction heterogeneity, prolongation of ventricular action potential duration via modulation of cardiac ion channels, impairment of intracellular Ca^2+^ handling proteins leading to spontaneous diastolic Ca^2+^ release, and enhanced cardiac sympathetic nervous system activation, collectively creating an arrhythmogenic substrate. Furthermore, they highlighted that elevated levels of these cytokines are independently associated with increased risk of ventricular arrhythmias and sudden cardiac death in various clinical settings, including patients with inflammatory diseases, coronary artery disease, and HF.^21^ Additionally, intestinal oedema and reduced nutrient absorption can further worsen systemic inflammation and immune dysfunction in advanced HF patients,^22^ contributing to arrhythmia risk.

In the subgroup analysis by prevention indication, the prognostic value of CONUT-defined malnutrition was significant in the primary prevention population but not in secondary prevention population. This difference has important clinical implications. In the secondary prevention population, arrhythmic risk is already established, potentially limiting the incremental prognostic contribution of CONUT score. Conversely, the primary prevention population is more heterogeneous, and the CONUT score may help to identify patients at particularly high arrhythmic risk. However, prospective studies are warranted to validate these subgroup findings.

Model discrimination for the primary outcome significantly improved (concordance index: 0.695 to 0.713, log-likelihood improvement: −3.54, *P* = .008) when CONUT-defined malnutrition was added to a base model (including the MADIT-ICD benefit score, LBBB, eGFR, natriuretic peptide levels, and history of ventricular arrhythmias). While the modest increase in concordance index is of limited value for individual patient risk stratification, this finding demonstrates that nutritional assessment captures prognostic information not fully reflected by these conventional clinical variables.

To evaluate the unique value of the CONUT score compared with other immuno-nutritional indices, we also performed additional analyses using the modified Glasgow Prognostic Score (mGPS), Prognostic Nutritional Index (PNI), and C-reactive protein/albumin ratio. Among these immuno-nutritional indices, the CONUT score demonstrated superior prognostic performance for predicting the composite outcome (ventricular arrhythmias or all-cause mortality) in the multivariate analysis (Supplementary Table S1).

These results suggest that nutritional assessment using CONUT scores may serve as a promising adjunctive risk-stratification tool in patients considered for CRT. Given the high AUC in the time-dependent ROC analysis (0.80 at 5 years), the CONUT score may offer incremental prognostic value beyond that of traditional risk factors. Although the MADIT-ICD benefit score is commonly used for CRT-D versus CRT-*P* selection, incorporating nutritional status may help refine the decision-making process.

### Limitations

This study had a few limitations. First, this was a retrospective single-centre analysis with a limited sample size, which may have affected the generalizability of our findings. Second, medication changes over time and the impact of nutritional interventions were not assessed. Finally, arrhythmic substrate characterization (e.g. scar imaging or electrophysiological mechanisms) was not available.

Future prospective studies are needed to validate our findings, explore the impact of nutritional interventions, and examine the mechanisms underlying malnutrition-associated arrhythmogenesis in patients with HF undergoing CRT.

## Conclusion

We found that malnutrition defined by a CONUT score of 5 or more was independently associated with an increased risk of ventricular arrhythmias and all-cause mortality among patients with advanced HF undergoing CRT. Therefore, CONUT-defined malnutrition may serve as a promising adjunctive risk-stratification tool in CRT recipients, particularly for primary prevention. However, prospective validation studies are required to confirm this.

## Supplementary Material

xvag037_Supplementary_Data

## Declarations

### Data Availability

The deidentified participant data will be shared on a request basis. Please directly contact the corresponding author to request data sharing.

### Pre-registered Clinical Trial Number

None supplied.
