# Supplementary material for: Controlling Nutritional Status score as a predictor of ventricular arrhythmias in patients with advanced heart failure
Source: ESC Heart Fail. 2026 Feb 26;13(1):xvag037. doi: 10.1093/eschf/xvag037 (PMC13108308; doi:10.1093/eschf/xvag037)
Supplement: xvag037_Supplementary_Data [file xvag037_supplementary_data.zip › R1_Supplementary Figure Legends_2025_10_8.docx]

**Supplementary Figure Legends**

Supplementary figure 1. The area under the curve (AUC) for CONUT-defined malnutrition in predicting the primary outcome was 0.80 (95%CI: 0.67-0.94).

CONUT, Controlling Nutritional Status.
