# Supplementary material for: Controlling Nutritional Status score as a predictor of ventricular arrhythmias in patients with advanced heart failure
Source: ESC Heart Fail. 2026 Feb 26;13(1):xvag037. doi: 10.1093/eschf/xvag037 (PMC13108308; doi:10.1093/eschf/xvag037)
Supplement: xvag037_Supplementary_Data [file xvag037_supplementary_data.zip › R1_Supplymentary_2025_10_8.docx]

Supplementary table1. Multivariate Cox proportional hazards analyses of clinical outcomes using other immuno-nutritional indices.

|  | **Univariate Cox regression analysis** | | **Multivariate Cox regression analysis** | | | |
| --- | --- | --- | --- | --- | --- | --- |
|  |  |  | **Model1** |  | **Model2** |  |
|  | **HR (95% CI)** | **p value** | **HR (95% CI)** | **P value** | **HR (95% CI)** | **P value** |
| **Using modified Glasgow Prognostic Score (mGPS)** |  |  |  |  |  |  |
| **Primary outcome; ventricular arrhythmias or all-cause mortality** | **1.79 (0.85 - 3.77)** | **0.12** | **1.63 (0.77 - 3.47)** | **0.20** | **1.78 (0.83 - 3.83)^a^** | **0.14** |
| **Secondary outcome; all-cause mortality** | **2.67 (1.27 - 5.63)** | **0.01** | **2.54 (1.11 - 5.84)** | **0.03** | **2.45 (1.05 - 5.68)^a^** | **0.04** |
| **Secondary outcome; ventricular arrhythmias** | **1.54 (0.54 - 4.37)** | **0.42** | **1.32 (0.46 - 3.76)** | **0.61** | **1.32 (0.45 - 3.85)^b^** | **0.61** |
|  |  |  |  |  |  |  |
| **Using Prognostic Nutritional Index (PNI)** |  |  |  |  |  |  |
| **Primary outcome; ventricular arrhythmias or all-cause mortality** | **1.63 (0.83 - 3.21)** | **0.16** | **1.84 (0.93 - 3.65)** | **0.08** | **1.84 (0.90 - 3.76)^a^** | **0.09** |
| **Secondary outcome; all-cause mortality** | **2.67 (1.27 - 5.63)** | **0.01** | **2.66 (1.24 - 5.70)** | **0.01** | **2.67 (1.19 - 5.99)^a^** | **0.02** |
| **Secondary outcome; ventricular arrhythmias** | **1.32 (0.51 - 3.41)** | **0.57** | **1.57 (0.60 - 4.06)** | **0.36** | **1.65 (0.62 - 4.38)^b^** | **0.31** |
|  |  |  |  |  |  |  |
| **Using CRP/Alb ratio** |  |  |  |  |  |  |
| **Primary outcome; ventricular arrhythmias or all-cause mortality** | **2.05 (1.21 - 3.48)** | **0.008** | **1.88 (1.10 - 3.22)** | **0.02** | **1.52 (0.88 - 2.62)^a^** | **0.14** |
| **Secondary outcome; all-cause mortality** | **2.16 (1.09 - 4.27)** | **0.03** | **1.93 (0.97 - 3.85)** | **0.06** | **1.87 (0.93 - 3.75)^a^** | **0.08** |
| **Secondary outcome; ventricular arrhythmias** | **2.00 (1.00 - 4.03)** | **0.05** | **1.86 (0.92 - 3.77)** | **0.09** | **1.17 (0.54 - 2.53)^b^** | **0.68** |

HR, hazard ratio; CI, confidence interval; CRP, C-reactive protein; Alb, albumin

Model 1: adjusted for age and sex.

Model 2a: adjusted for left bundle branch block (LBBB), estimated glomerular filtration rate (eGFR), median brain natriuretic peptide (BNP), N-terminal pro-B-type natriuretic peptide (NT-proBNP) levels, and history of ventricular arrhythmias.

Model 2b: adjusted for age, sex, LBBB, median BNP, NT-proBNP level, and history of ventricular arrhythmias

Modified Glasgow Prognostic Score (mGPS): CRP >1.0 mg/dL and albumin <3.5 g/dL = 2; CRP >1.0 mg/dL and albumin ≥3.5 g/dL = 1; CRP ≤1.0 mg/dL = 0. Malnutrition defined as mGPS ≥1.

Prognostic Nutritional Index (PNI): calculated as 10 × serum albumin (g/dL) + 0.005 × total lymphocyte count (/μL). Malnutrition defined as PNI <38.

CRP/Albumin ratio: CRP (mg/dL) divided by albumin (g/dL). Malnutrition defined as high CRP/Alb ratio (≥0.031) based on the median value.
