# Supplementary figures and images for: Controlling Nutritional Status score as a predictor of ventricular arrhythmias in patients with advanced heart failure
Source: ESC Heart Fail. 2026 Feb 26;13(1):xvag037. doi: 10.1093/eschf/xvag037 (PMC13108308; doi:10.1093/eschf/xvag037)

## Slide 1
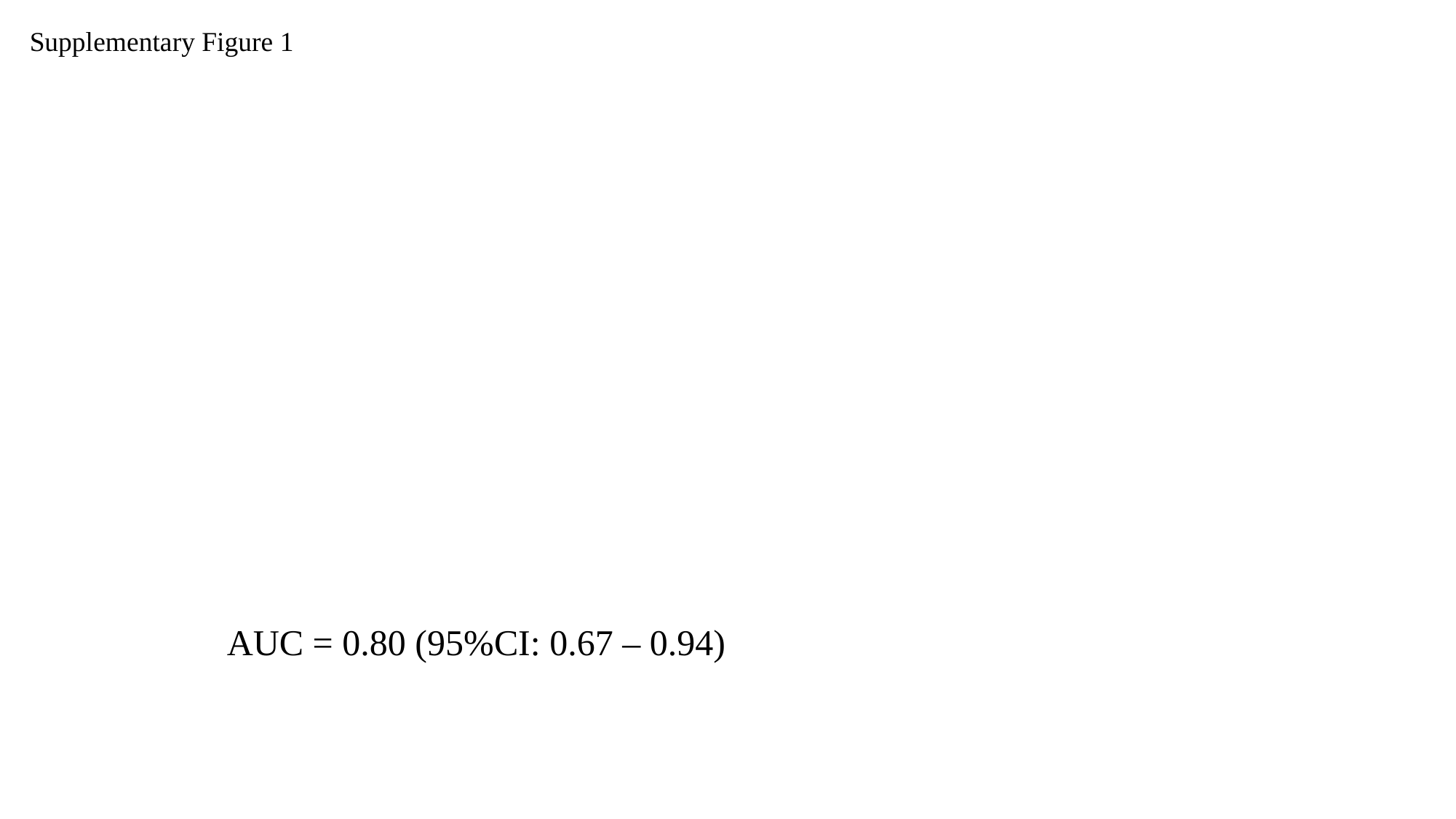

Supplementary Figure 1
AUC = 0.80 (95%CI: 0.67 – 0.94)

Supplement: xvag037_Supplementary_Data [file xvag037_supplementary_data.zip › R1_CONUT supplymentary figure_2025_10_8.pptx]
